# Complete twelve month bone remodeling with a bi-phasic injectable bone substitute in benign bone tumors: a prospective pilot study

**DOI:** 10.1186/s12891-015-0828-3

**Published:** 2015-11-26

**Authors:** Jacek Kaczmarczyk, Piotr Sowinski, Maciej Goch, Katarzyna Katulska

**Affiliations:** Department of Orthopedics and Traumatology, Poznań University of Medical Sciences, Poznan, Poland; Department of Radiology, Poznań University of Medical Sciences, Poznan, Poland

**Keywords:** Bone substitute, Synthetic, Benign, Bone cyst, Ceramic, Injectable, Bone remodelling, Minimal invasive, Prospective, Humans

## Abstract

**Background:**

Benign primary bone tumors are commonly treated by surgery involving bone grafts or synthetic bone void fillers. Although synthetic bone grafts may provide early mechanical support while minimizing the risk of donor-site morbidity and disease transmission, difficult handling properties and less than optimal transformation to bone have limited their use.

**Methods:**

In a prospective series, patients with benign bone tumors were treated by minimal invasive intervention with a bi-phasic and injectable ceramic bone substitute (CERAMENT™ BONE VOID FILLER, BoneSupport, Sweden) with the hypothesis that open surgery with bone grafting might be avoided. The defects were treated by either mini-invasive surgery (solid tumors) or percutaneous injection (cysts) and followed clinically and radiologically for 12 months. CT scan was performed after 12 months to confirm bone remodeling of the bone substitute. All patients were allowed full weight bearing immediately after surgery.

**Results:**

Fourteen patients with a median age of 13 years (range 7–75) were consecutively recruited during 11 months. Eleven lesions were bone cysts (eight unicameral and three post-traumatic) and three were solid benign tumors. The median size of the lesions was 40 mL (range 1–152). The most common location was humerus (*n* = 10). After 12 months the defects completely or partially filled with median 18 mL (range 5–28) of bone substitute demonstrated full resolution (Neer Classification grade I) in 11 patients, partial resolution (Neer II) in 2 patients and in 1 patient the cyst persisted (Neer III).

No lesions required recurrent surgery during the observation period. No post-operative fracture or infection was recorded.

**Conclusions:**

Minimal invasive treatment with a bi-phasic and injectable ceramic bone substitute might offer an alternative to regular bone grafting due to convenient handling properties and rapid bone remodeling.

**Trial Registration:**

ClinicalTrials NCT02567084 Release Date 10/01/2015

## Background

Benign bone tumors are often treated with intralesional curettage which creates a bone defect that can be filled with e.g. demineralized bone matrix, autologous bone, ceramic bone substitutes or polymethylmetacrylate cement [1–4].

Autograft has been considered the golden standard because it possesses all three of the essential elements required for an optimal bone graft [5], but is associated with morbidity at the donor site and is limited in supply [6]. Allograft has been employed as a good alternative to autograft but the concern for potential disease transmission remains [7]. Synthetic bone graft substitutes have been gaining popularity as viable alternatives for void and defect filling eliminating the concerns with autograft and allograft. These synthetic bone substitutes have invariably been based on calcium phosphate and/or calcium sulfate materials which are osteoconductive and facilitate bone remodeling, although either side effects such as drainage and wound complications [8, 9] slow remodeling to bone [10, 11] or negligible bone generation [9, 12] have limited their use. Thus, new synthetic bone substitutes with described positive effects in vertebroplasty [13, 14], osteotomy [15], and smaller trauma defects [16] merit further investigation also in treatment of larger bone defects.

Presented here are the 12 months radiological and functional results of a prospective patient series of benign bone tumors treated with a rapidly remodeling bi-phasic injectable ceramic bone substitute [17] applied either percutaneously or through mini-invasive surgery, with the hypothesis that open surgery and bone grafting might be avoided with acceptable clinical outcome.

## Methods

### Materials

A bi-phasic ceramic bone substitute (CERAMENT™ BONE VOID FILLER, Bone Support AB, Lund, Sweden) composed of 60 % weight synthetic calcium sulfate (CaS) and 40 % weight hydroxyapatite (HA) powder was mixed with a water-soluble radio-contrast agent iohexol (180 mg/ml) to make the material radiopaque. The combination produces an easily injectable paste that sets within 15 min and fully hardens after 60 min and shows a wet compressive strength exceeding that of healthy cancellous bone [18]. The CaS component undergoes gradual reabsorption during the first months being replaced by in-growing bone that remodels to form trabecular bone whereas the HA component has a slower reabsorbing rate and is incorporated into the newly formed trabecular bone [17].

### Patient handling

Fourteen consecutive patients fulfilling the criteria of having a benign bone lesion requiring bone grafting after surgery were included. Exclusion criteria were known hyperthyroidism or autonomous thyroid adenoma, history of serious reaction to iodine based radio-contrast agents, local infection at the site of implantation, pre-existing calcium metabolism disorder (e.g. hypercalcemia), or a bleeding disorder.

The volume of defects was calculated on plain radiograms as: width/2 × depth/2 × height × π for defects of cylindrical form and width/2 × depth/2 × height/2 × 4/3π for spherical defects [19].

Bone cysts of a volume exceeding 100 cc were treated with an injection of 1–2 mL of methyl prednisolone 40 mg/mL (Depot Medrol®, Pfizer) in an attempt to induce bone generation and reduce the cyst volume. When further reduction of the cyst volume was needed, 1 or 2 repeated injections were performed at an interval of 6 months [20].

Due to our lack of experience in injecting the actual product at volumes bigger than 30 cc, and after consultation with the manufacturer, this amount was set as the maximum treatment volume in spite of some defects being bigger. All patients were assessed pre-operatively to calculate the volume of the defect and to decide whether the entire defect could be filled with the bone substitute or if critical parts of the defect should be prioritized. Critical parts were defined as regions within the defect considered by the surgeon to be at highest risk for fracture. Full weight bearing was allowed immediately after surgery. All patients underwent radiographic examination with X-ray pre-operatively, post-operatively and after 3 and 12 months, and 11 patients underwent CT scan after 12 months to investigate possible bone bridging of the defect. All patients were giving their informed consent written and the study was approved by Ethics Committee of Medical University im. Karola Marcinkowskiego in Poznan Collegium Maiusul. Fredry 10 61–701 Poznań Local Ethics Committee. The research reported in the paper was undertaken in compliance with the Helsinki Declaration.

### Surgical technique

**Fig. 1:**
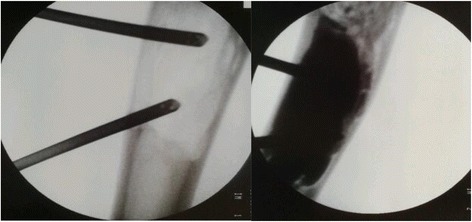
Two needles technique. Transcortical administration of the flowable bone substitute by the use of one injection needle and one “ventilation” needle. Before (*left*) and immediately after (*right*) injection of the bone substitute

All procedures were performed under general anesthesia guided by fluoroscopy. To minimize the risk of soft tissue leakage a mini-invasive technique was used. Solid benign tumors were removed through a cortical bone window big enough to allow biopsies for histopathological examination and to ensure radical excision. For bone cysts a completely closed technique was used. An 11 gauge bone needle was introduced transcortically under fluoroscopy into the proximal part of the cyst to act as a ventilation needle, and another needle was similarly placed at the distal end (Fig. 1). Such a two-needle-system provides more control and efficiency in cyst filling or aspiration due to low injection or aspiration pressure. The cyst fluid was then aspirated followed by repeated flushing with 0.9 % saline. In the case of single bone membranes the needle was forced through the bone to ensure best possible communication. To enable bone remodeling of the subsequently injected bone substitute proper contact with cancellous bone was ensured by spot-wise scratching of the epithelial lining with the tip of the needle until bleeding was demonstrated in the saline.

**Fig. 2:**
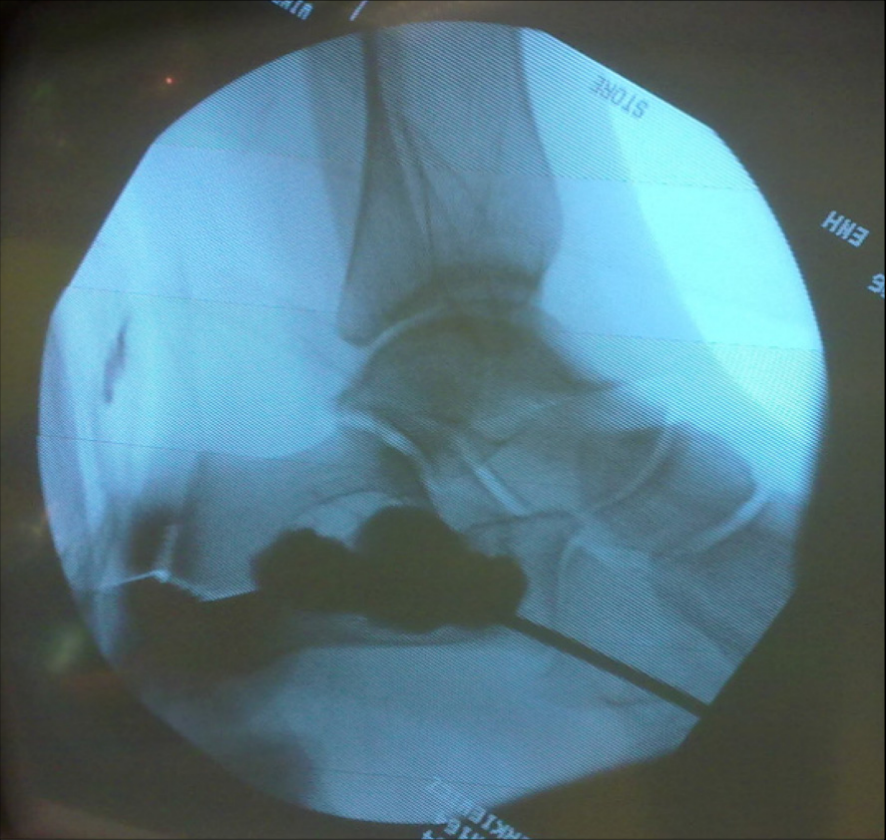
Transcortical injection. The bone graft material injected through an 11G bone needle into a calcaneal bone cyst under fluoroscopic guidance. Note the high radiovisibility caused by the addition of water-soluble radio-contrast agent

The bone substitute paste was then prepared by mixing the liquid component containing iohexol with the powder for 30 s. At time = 4 min. from the start of mixing the paste was sufficiently viscous to withstand dissolution by the rinse fluid and injection was started through the distal needle with the opposite needle allowing passive evacuation of the fluid. Due to high content of iohexol the entire injection could be easily followed under fluoroscopy (Fig. 2). Although the aim was to completely fill the cyst, this was far from always possible due to interfering bone membranes and excessive cyst volume. After completed filling, stylets were introduced into the needles and left untouched until time = 7 min. When resistance was felt at slight pulling of the needle the two needles were removed by a rotating movement.

Bone healing was independently evaluated using the modified Neer score system [1] by an external physician being a specialist in orthopedic radiology. The classification is shown in Table 1. CT scan was used in a qualitative way to demonstrate bone bridging of the defects and absence of remaining bone substitute, as an indirect sign of bone remodeling. All patients received standard post-operative treatment with paracetamol 1 g 4 times daily.

**Table 1:** Modified Neer classification of radiologic results

Score	Classification	Description	Treatment
I	Healed	Cyst filled with new bone, with or without small radiolucent area(s) < 1 cm in size	Not necessary
II	Healed with defects	Radiolucent area(s) < 50 % of the diameter of the bone with enough cortical thickness to prevent fracture	Not necessary
III	Persistent cyst	Radiolucent area > 50 % of the diameter of the bone and with a thin cortical rim; no increase of the size of the cyst	Continued restriction of activity, possible repeated treatment required
IV	Recurrent or nonresponsive cyst	Cyst reappeared in a previously obliterated area or a radiolucent area has increased in size	Need for repeated treatment

## Results

Fourteen patients were consecutively recruited at one center during 11 months and followed for 12 months without any drop-out. The median age was 13 years with a range between 7 and 75 years. Eight patients were 14 or younger. Eight lesions were unicameral, three were post-traumatic bone cysts and other three patients had a solid benign lesion. The most common lesion location was the humerus (*n* = 10). The median volume of the lesions was 40 mL (range 1–152). The individual demographic data and description of the bone lesions are shown in Table 2.

**Table 2:** Details of patients

No.	Symptom	Location	Diagnosis	Size (cm^3^)
1	Pain	Finger	Enchondroma	1
2	Pain	Humerus	UBC	62
3	Fracture	Humerus	Post-traumatic	152
4	Pain	Humerus	UBC	24
5	Pain	Humerus	Chondroblastoma	114
6	Pain	Femur	Fibrous defect	23
7	Pain	Calcaneus	UBC	49
8	Pain	Humerus	UBC	70
9	Pain	Humerus	UBC	37
10	Fracture	Humerus	Post-traumatic	38
11	Fracture	Humerus	Post-traumatic	42
12	Pain	Humerus	UBC	46
13	Pain	Humerus	UBC	34
14	Pain	Fibula	UBC	16

Patient demographics and description of the bone lesions

*UBC* Unicameral Bone Cyst

**Fig. 3:**
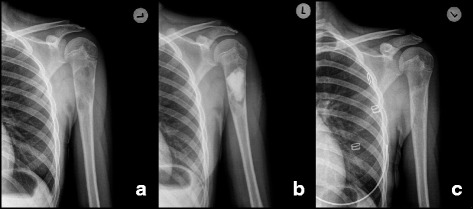
Full remodeling after 12 months. Patient treated with 15 mL of bone substitute. Pre-op (**a**), post-op (**b**) and 12 months follow-up (**c**)

**Fig. 4:**
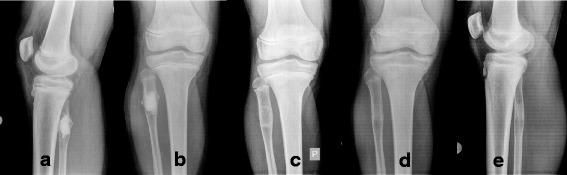
Full remodeling after 12 months. Patient with a unicameral bone cyst mini-invasively injected with the synthetic none substitute. Immediately postop (**a**, **b**), at 3 months (**c**), and after 12 months (**d**, **e**)

**Fig. 5:**
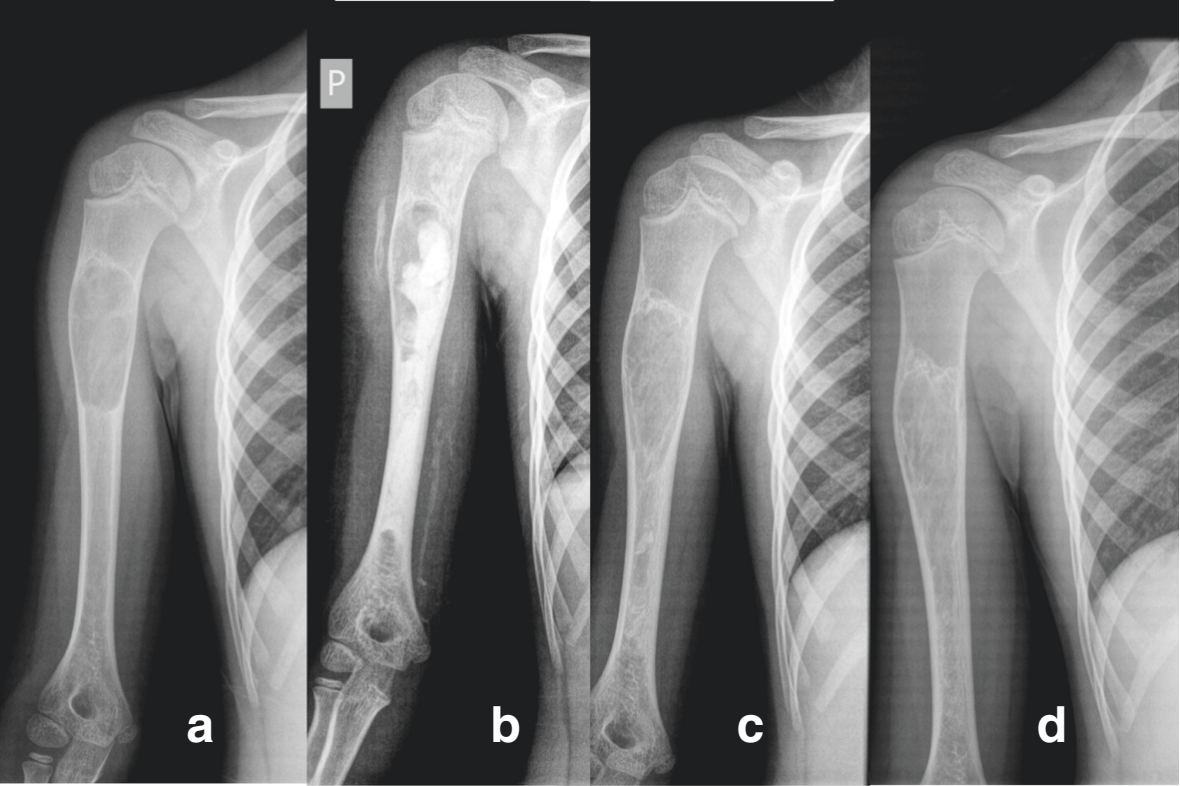
Full remodeling in spite of incomplete filling. Patient mini-invasively treated with 18 mL of the injectable bone substitute; Pre-op (**a**). In spite of incomplete filling (**b**) a general bone formation process is triggered, which can be seen at 3 months (**c**) and with complete radiological remodeling at 12 months (**d**)

**Fig. 6:**
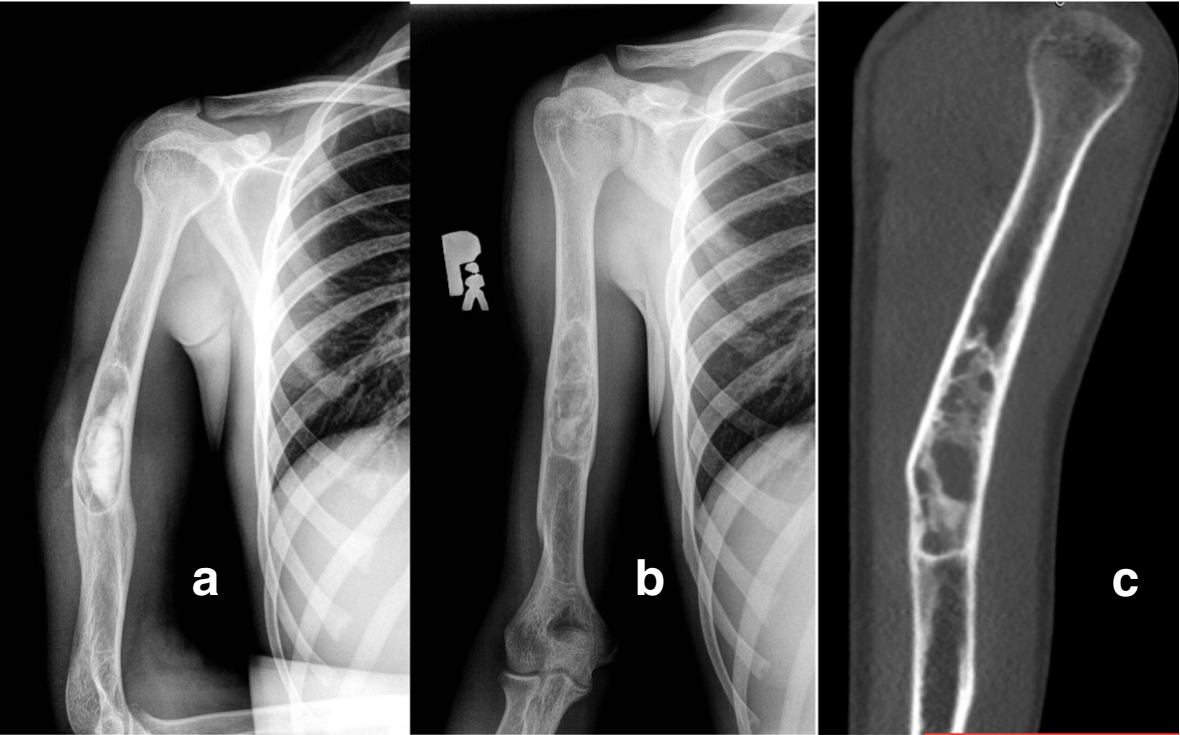
Twelve months follow-up with CT scan. Patient treated with 18 mL of CERAMENT™ due to a post-traumatic bone cyst. X-ray immediately post-op. **a** and after 12 (**b**) months. CT scan after 12 months demonstrating trabecular bone bridging (**c**)

Two patients were treated with repeated injections of methyl prednisolone (patient 3 and 5) with marginal effect. The median volume of bone substitute used was 18 mL (range 5–28). Postoperative drainage lasted for 1 day (range 1–5). After 12 months full cyst resolution (Neer Classification grade I) was seen in 11 patients (Figs. 3 and 4) despite incomplete filling (Fig. 5) in majority of cases (Table 3). Partial healing (Neer II) was demonstrated in 2 patients and in one patient the cyst persisted (Neer III). A CT scan after 12 months confirmed the complete radiological transformation of the bone substitute into bone (Fig. 6). No post-operative infections or fractures were recorded during the 12 months follow-up period. All patients were free from pain within 10 days, and none required extra pain medication or treatment with non-steroid anti-inflammatory drugs.

**Table 3:** Treatment outcome

Patient No.	1	2	3	4	5	6	7	8	9	10	11	12	13	14
Size (cm^3^)	1	62	152	24	114	23	49	70	37	38	42	46	34	16
CERAMENT™ injected (mL)	1	18	18	15	18	28	23	18	18	10	8	18	18	10
Bone healing (Neer classification)	I	I	II	I	I	I	II	III	I	I	I	I	I	I

## Discussion

The presented prospective patient series shows a new bone remodeling technique that relies on the properties ascribed to the ceramic bone substitute composite used [17]. The immediate pain relief, full weight-bearing recovery, bone consolidation and incorporation as well as the structural bone integrity and durability demonstrated in the study indicate that the material can provide a safe and effective long-term solution for the treatment of benign bone tumors. Of special interest is the ability of percutaneous transcortical injection under fluoroscopy in patients with juvenile cysts, which is enabled by mixing the ceramic powder composition with the water soluble radiocontrast agent iohexol.

An interesting observation was that the epithelium of unicameral cysts did not require complete removal since multiple scratching with the needle tip was sufficient to allow fresh bone to come in contact with the ceramic implant and provide full remodeling to bone. Another observation was that in spite of incomplete filling complete or almost complete healing was attained, which indicates that the material or the procedure triggers a bone healing process beyond that facilitated by the material itself [19].

The biphasic ceramic bone substitute is designed to remodel in tune with the natural bone remodeling process. Due to the microporosity of the cured calcium sulfate component an immediate flow of tissue fluids with nutrients and growth factors is allowed so to penetrate the implant. That in turn promotes osteoclasts and macrophages to enter the material and create macropores resulting in a widespread ingrowth of early bone [17]. The end result seems to be full transformation and remodeling into mature bone after 9–12 months [13, 15, 17]. In a previous study on a composite bone graft substitute similarly consisting of calcium sulfate and hydroxyapatite (Schindler et al.) the graft material was still present after two years, whereas in the current study a CT-confirmed complete bone remodeling was demonstrated on X-ray after one year in all but one patient. One explanation for the faster remodeling in the present study might be a more favorable proportion of calcium sulfate to hydroxyapatite (CaS/HA = 60 to 40 %) compared to that used in the study by Schindler and co-workers [21] where the graft material consisted of somewhat inverted proportion of 35 to 65 %. Reasonably fast remodeling has also been described with composite calcium phosphate based products [11, 22].

It has been reported that products consisting of pure calcium sulfate have a tendency to quickly dissolve [12] which, together with a low pH [23], leads to a high frequency of long term drainage [8] and subsequent wound complications [9]. Also, products mainly based on calcium phosphate cements have been reported to cause adverse and sometimes painful soft tissue reactions [24, 25]. The product used in the study presents with a neutral pH [17] and once implanted a passive precipitation of endogenous hydroxyapatite takes places on the implant surface [26], which seems to extend the gradual resorption of the calcium sulfate component over months [13] allowing the cement to resist immediate dissolution and be actively degraded and replaced by ingrowing bone that eventually remodels to form trabeculae [18, 27]. These two product differences (pH and composition) might partly explain: 1) the absence of prolonged post-operative drainage and/or late wound complications, 2) the rapid and reliable remodeling to bone [15]. The minimal invasive technique might also have contributed to the absence of adverse reactions due to less leakage into the soft tissue. Although the actual prospective study presents with favorable results, it must be emphasized that it is a rather small and non-controlled study which requires repeated and larger trials to confirm the findings.

## Conclusions

The investigated bi-phasic bone substitute seems to be a material with attractive handling properties including reliable injectability, enhanced radio-opacity and fast remodeling to bone, which might make it an attractive alternative to autologous bone or allografts.

## Abbreviations

CaS: Calcium sulphate
CT: Computerized tomography
HA: Hydroxyapatite

## References

[1] Cho SC, Seo HS, Park SH, Park JH, Shin DS, Park IH (2012). Minimal invasive surgery for unicameral bone cyst using demineralized bone matrix: a case series. BMC Musculoskelet Disord.

[2] Bickels J, Meller I, Shmookler BM, Malawer MM (1999). Review article. The role and biology of cryosurgery in the treatment of bone tumors. Acta Orthop Scand.

[3] Mik G, Arkader A, Manteghi A, Dormans JP (2009). Results of a minimally invasive technique for treatment of unicameral bone cysts. Clin Orthop Relat Res.

[4] Campanacci M, Capanna R, Fabbri N, Bettelli G (1999). Curettage of giant cell tumor of bone. Reconstruction with subchondral grafts and cement. Chir Organi Mov.

[5] Khan SN, Tomin E, Lane JM (2000). Clinical applications of bone graft substitutes. Orthop Clin North Am.

[6] Myeroff C, Archdeacon M (2011). Autogenous bone graft: donor sites and techniques. J Bone Joint Surg Am.

[7] Hinsenkamp M, Muylle L, Eastlund T, Fehily D, Noël L, Strong DM (2012). Adverse reactions and events related to musculoskeletal allografts: reviewed by the World Health Organisation Project NOTIFY. Int Orthop.

[8] Lee GH, Khoury JG, Bell J-E, Buckwalter JA (2002). Adverse reactions to Osteoset bone graft substitute. The incidence in a consecutive series. Iowa Orthop J.

[9] Ferguson JY, Dudareva M, Riley ND, Stubbs D, Atkins BL, McNally MA (2014). The use of a biodegradable antibiotic-loaded calcium sulphate carrier containing tobramycin for the treatment of chronic osteomyelitis: a series of 195 cases. Bone Joint J.

[10] Reppenhagen S, Reichert JC, Rackwitz L, Rudert M, Raab P, Daculsi G (2012). Biphasic bone substitute and fibrin sealant for treatment of benign bone tumors and tumor-like lesions. Int Orthop.

[11] Fillingham YA, Lenart BA, Gitelis S (2012). Function after injection of benign bone lesions with a bioceramic. Clin Orthop Relat Res.

[12] Petruskevicius J, Nielsen S, Kaalund S, Knudsen PR, Overgaard S (2002). No effect of Osteoset, a bone graft substitute, on bone healing in humans: a prospective randomized double-blind study. Acta Orthop Scand.

[13] Hatten HP, Voor MJ (2012). Bone healing using a bi-phasic ceramic bone substitute demonstrated in human vertebroplasty and with histology in a rabbit cancellous bone defect model. Interv Neuroradiol.

[14] Masala S, Nano G, Marcia S, Muto M, Fucci FP, Simonetti G (2012). Osteoporotic vertebral compression fracture augmentation by injectable partly resorbable ceramic bone substitute (Cerament™|SPINESUPPORT): a prospective nonrandomized study. Neuroradiology.

[15] Abramo A, Geijer M, Kopylov P (2010). Osteotomy of distal radius fracture malunion using a fast remodelling bone substitute consisting of calcium sulfate and calcium phosphate. J Biomed Mater Res B Appl Biomater.

[16] Nusselt T, Hofmann A, Rommens PM (2013). The injectable biphasic calcium sulphate/hydroxyapatite bone substitute CERAMENT™ possesses reliable remodeling activity in metaphyseal fracture defects [abstract]. Eur J Trauma Emerg Surg.

[17] Nilsson M, Zheng MH, Tagil M (2013). The composite of hydroxyapatite and calcium sulfate: a review of preclinical evaluation and clinical applications. Expert Rev Med Devices.

[18] Wang JS, Zhang M, McCarthy I, Tanner KE, Lidgren L. Biomechanics and bone integration on injectable calcium sulfate and hydroxyapatite in large bone defect in rat [abstract], ORS 2006. http://www.ors.org/Transactions/52/0879.pdf. (02/12/2014).

[19] Hirn M, de Silva U, Sidhartan S, Grimer RJ, Abudu A, Tillman RM (2009). Bone defects following curettage do not necessarily need augmentation. Acta Orthop.

[20] Sung AD, Anderson ME, Zurakowski D, Hornicek FJ, Gebhardt MC (2008). Unicaneral bone cyst. A retrospective study on three surgical techniques. Clin Orthop Relat Res.

[21] Schindler OS, Cannon SR, Briggs TWR, Blunn GW (2008). Composite ceramic bone graft substitute in the treatment of locally aggressive benign bone tumours. J Orthop Surg.

[22] Evaniew N, Tan V, Parasu N, Jurriaans E, Finlay K, Deheshi B (2013). Use of a calcium sulfate-calcium phosphate synthetic bone graft composite in the surgical management of primary bone tumors. Orthopedics.

[23] Walsh WR, Morberg P, Yu Y, Yang JL, Haggard W, Sheath PC (2003). Response of a calcium sulfate bone graft substitute in a confined cancellous defect. Clin Orthop Relat Res.

[24] Welkerling H, Raith J, Kastner N, Marschall C, Windhager R (2003). Painful soft-tissue reaction to injectable Norian SRS calcium phosphate cement after curettage of enchondromas. J Bone Joint Surg (Br).

[25] Friesenbichler J, Maurer-Ertl W, Sadoghi P, Pirker-Fruehauf U, Bodo K, Leithner A (2014). Adverse reactions of artificial bone graft substitutes: Lessons learned from using tricalcium phosphate geneX. Clin Orthop Relat Res.

[26] Nilsson M, Wang JS, Wielanek L, Tanner KE, Lidgren L (2004). Biodegradation and biocompatibility of a calcium sulfate-hydroxyapatite bone substitute. J Bone Joint Surg [Br].

[27] Mahan KT, Hillstrom HJ (1998). Bone grafting in foot and ankle surgery. A review of 300 cases. J Am Pod Med Assoc.

